# p100 Deficiency Is Insufficient for Full Activation of the Alternative NF-κB Pathway: TNF Cooperates with p52-RelB in Target Gene Transcription

**DOI:** 10.1371/journal.pone.0042741

**Published:** 2012-08-06

**Authors:** Agnes Lovas, Anja Weidemann, Daniela Albrecht, Lars Wiechert, Debra Weih, Falk Weih

**Affiliations:** 1 Research Group Immunology, Leibniz-Institute for Age Research – Fritz-Lipmann-Institute, Jena, Germany; 2 Biocontrol Jena GmbH, Jena, Germany; 3 Faculty of Biology and Pharmacology, Friedrich-Schiller-University, Jena, Germany; Sun Yat-sen University Medical School, China

## Abstract

**Background:**

Constitutive activation of the alternative NF-κB pathway leads to marginal zone B cell expansion and disorganized spleen microarchitecture. Furthermore, uncontrolled alternative NF-κB signaling may result in the development and progression of cancer. Here, we focused on the question how does the constitutive alternative NF-κB signaling exert its effects in these malignant processes.

**Methodology/Principal Findings:**

To explore the consequences of unrestricted alternative NF-κB activation on genome-wide transcription, we compared gene expression profiles of wild-type and NF-κB2/p100-deficient (*p100^−/−^*) primary mouse embryonic fibroblasts (MEFs) and spleens. Microarray experiments revealed only 73 differentially regulated genes in *p100^−/−^* vs. wild-type MEFs. Chromatin immunoprecipitation (ChIP) assays showed in *p100^−/−^* MEFs direct binding of p52 and RelB to the promoter of the *Enpp2* gene encoding ENPP2/Autotaxin, a protein with an important role in lymphocyte homing and cell migration. Gene ontology analysis revealed upregulation of genes with anti-apoptotic/proliferative activity (*Enpp2/Atx*, *Serpina3g*, *Traf1*, *Rrad*), chemotactic/locomotory activity (*Enpp2/Atx*, *Ccl8*), and lymphocyte homing activity (*Enpp2/Atx*, *Cd34*). Most importantly, biochemical and gene expression analyses of MEFs and spleen, respectively, indicated a marked crosstalk between classical and alternative NF-κB pathways.

**Conclusions/Significance:**

Our results show that p100 deficiency alone was insufficient for full induction of genes regulated by the alternative NF-κB pathway. Moreover, alternative NF-κB signaling strongly synergized both *in vitro* and *in vivo* with classical NF-κB activation, thereby extending the number of genes under the control of the p100 inhibitor of the alternative NF-κB signaling pathway.

## Introduction

Activation of nuclear factor-κB (NF-κB) plays a central role in regulation of innate and adaptive immunity, cell proliferation, apoptosis, cancer, and lymphoid organ development [1], [2], [3], [4], [5]. In vertebrate cells, this pivotal transcription factor family is comprised of two groups: the Rel and NF-κB proteins. Whereas the members of the first (RelA, RelB, c-Rel) are synthesized as mature forms, members of the latter, p50 and p52, are synthesized as precursors p105/NF-κB1 and p100/NF-κB2, respectively. These structurally related proteins share similar sequences within their N-terminal Rel homology domain (RHD) that enables them to dimerize, to translocate into the nucleus, and to bind to specific DNA sequences named κB sites. However, only RelA, RelB and c-Rel contain a C-terminal transcriptional activation domain and therefore are able to directly activate transcription. The other two members (p105 and p100) harbor an inhibitory domain comprising ankyrin repeats in their C-termini. Proteasomal processing results in the removal of this inhibitory domain and p50 and p52 together with their dimerization partners RelA, RelB, or c-Rel can act as transcriptional activators. In addition, homodimerization of p50 or p52 leads to transcriptionally inactive nuclear complexes. However, association with nuclear Bcl3 converts p52-p52 or p50-p50 homodimers into transcriptionally active p52-p52-Bcl3 or p50-p50-Bcl3 heterotrimers [6], [7], [8].

In most cells, NF-κB complexes are maintained latent in the cytoplasm in association with inhibitors of the IκB family. A wide range of stimuli, such as cytokines, lipopolysaccharides (LPS), DNA damaging agents, Toll-like receptor agonists, or viruses, induce the so-called classical NF-κB pathway as characterized by the nuclear translocation of predominantly p50-RelA complexes. The liberation of p50-RelA from its cytoplasmic IκB inhibitors depends on the activation of the IKK (inhibitor of κB kinase) complex. IKKs phosphorylate IκB molecules, resulting in their ubiquitination, proteasomal degradation, and translocation of p50-RelA into the nucleus. In turn, p50-RelA regulates numerous target genes that are predominantly involved in innate immunity, cell survival, and inflammation. A few inducers of the classical NF-κB pathway, such as LTα_1_β_2_, CD40L, BAFF, TWEAK, and RANKL, are able to trigger an additional pathway through the activation of the NF-κB-inducing kinase (NIK) and IKKα. This pathway has been named alternative or non-canonical NF-κB pathway and is characterized by the post-translational processing of p100 to the mature p52 subunit. In most unstimulated cells folding of the C-terminal ankyrin repeats domain masks the nuclear localization signal (NLS) of p100, which therefore is mainly cytosolic. IKKα phosphorylates p100 in a stimulus-dependent manner, resulting in p100 ubiquitination and its subsequent proteasomal processing to p52. p100 is the main inhibitor of RelB and nuclear p52-RelB complexes control genes that are predominantly involved in adaptive immunity and lymphoid organ development [9], [10], [11], [12], [13], [14], [15], [16].

Under physiological conditions, activation of the alternative pathway is under the control of members of the TNFR (tumor necrosis factor receptor) family, which are involved in secondary lymphoid organ development, B cell survival and homeostasis, as well as osteoclastogenesis (LTβR, CD40, BAFFR, RANK). Thus, regulated production of p52 is important for a robust progression of these processes [10], [17], [18], [19]. However, several lines of evidence show that uncontrolled p52 expression can also lead to pathology and to development and progression of cancer. For our studies, we have used the *p100^−/−^* mouse model generated by Ishikawa and coworkers [20] in which p52 is overexpressed in the absence of p100. These mice suffer from *i)* gastric hyperplasia resulting in early postnatal death, *ii)* enlarged lymph nodes, *iii)* increased proliferative responses of lymphocytes, *iv)* accumulation of granulocyte precursors and neutrophils in the bone marrow [20], *v)* disruption of the marginal sinus combined with ectopic high endothelial venules in the red pulp of the spleen [21], and *vi)* impaired early B cell development in the bone marrow [22].

Moreover, tumor-associated truncations of p100 have transforming effects in murine fibroblasts [23]. In humans, chromosomal translocations that cause *NFKB2* gene rearrangements and constitutive processing of p100 lead to B and T cell lymphomas [17], [18]. Deregulated p100 processing has also been associated with T cell transformation by the human T cell leukemia virus type I [24] and constitutive NF-κB activation in breast and prostate cancer cell lines [25], [26].

Here, we investigated the consequences of constitutive alternative NF-κB activation on genome-wide transcription. To achieve this, we compared gene expression profiles of wild-type and NF-κB2/p100-deficient (*p100^−/−^*) primary mouse embryonic fibroblasts (MEFs). Surprisingly, we found that p100 deficiency alone was insufficient for full induction of genes that are regulated by the alternative NF-κB pathway. Our data demonstrate that alternative NF-κB signaling strongly synergized both *in vitro* and *in vivo* with classical NF-κB activation, thereby extending the number of genes under the control of the p100 inhibitor of the alternative NF-κB pathway. This strong crosstalk between the two NF-κB pathways indicates that uncontrolled alternative NF-κB signaling was further enhanced by classical NF-κB activation.

## Results and Discussion

**Figure 1:**
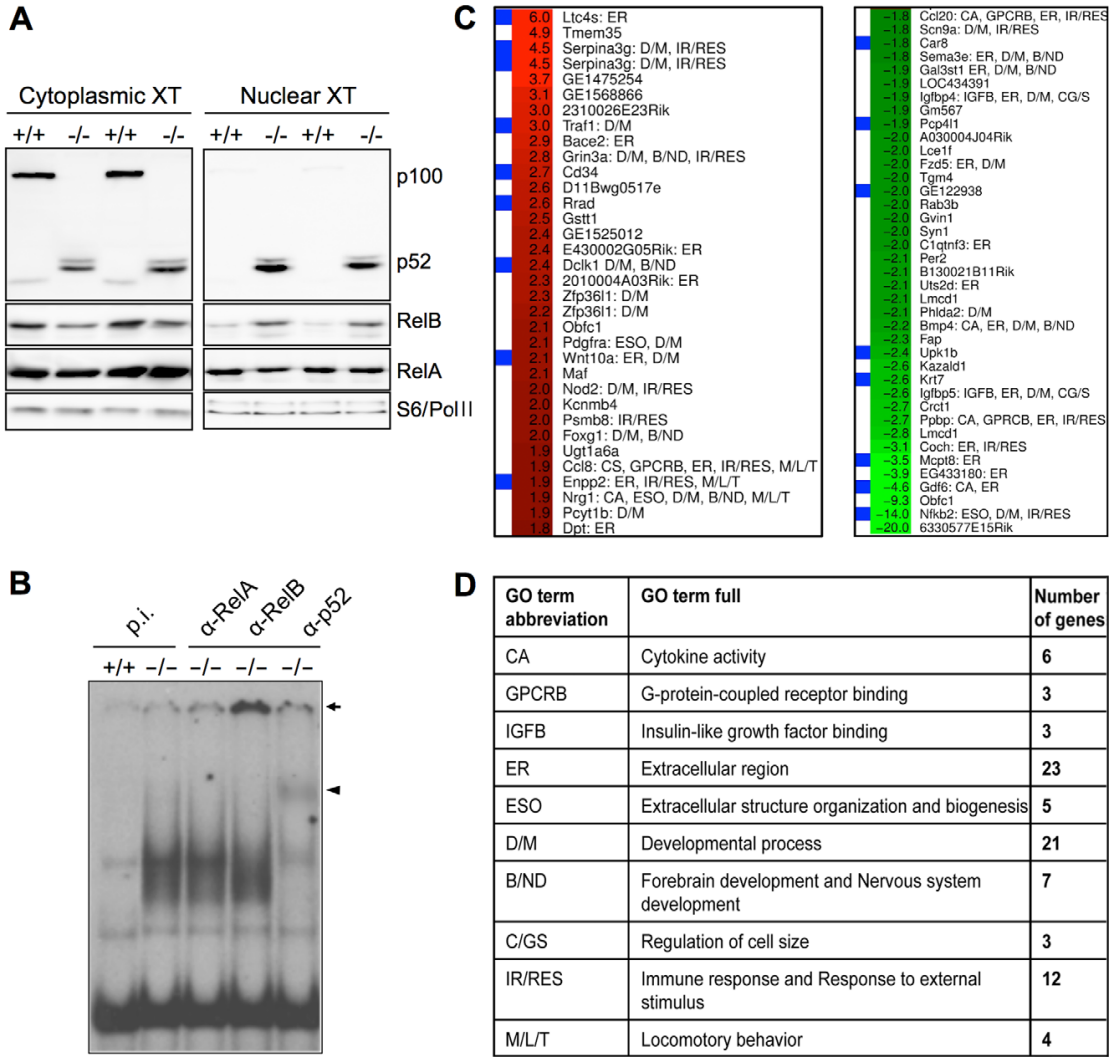
Expression profiling of wild-type and *p100^−/−^* MEFs. (A) Western blot analysis of cytoplasmic and nuclear protein extracts (20 µg/sample) were analyzed for the presence of NF-κB family members p100/p52, RelB, and RelA in wild-type (+/+) and in *p100^−/−^* (−/−) MEFs. As cytoplasmic loading control S6 ribosomal protein and as nuclear loading control RNA Pol II was assayed. (B) Increased κB DNA-binding activity in nuclear extracts from *p100^−/−^* (−/−) compared to wild-type MEFs (+/+). Five µg protein extract per cell line were incubated with a radioactively labeled Igκ oligo and analyzed by EMSA. Supershift analysis was performed using pre-immune serum (p.i.), anti-RelA (α-RelA), anti-RelB (α-RelB), and anti-p52 antibodies (α-p52). Super-shifted RelB and p52 complexes are indicated by arrow and arrowhead, respectively. (C) Heatmap displaying fold change values observed in *p100^−/−^* vs. wild-type cells. The color code indicates the fold change values between +6 fold up- (red) and −20 fold downregulation (green). Each horizontal line on the heatmap corresponds to one gene. Genes labeled with blue boxes on the left were verified by qRT-PCR. Gene symbols and abbreviations of GO terms are displayed on the right. CA, Cytokine activity; GPCRB, G-protein-coupled receptor binding; IGFB, insulin-like growth factor binding; ER, extracellular region; ESO, extracellular structure organization and biogenesis; D/M, developmental process; B/ND, forebrain development and nervous system development; CG/S, regulation of cell size; IR/RES, immune response and response to external stimulus; M/L/T, locomotory behavior. (D) Significantly regulated Gene Ontology terms with respective gene numbers.

To explore the consequences of constitutively active alternative NF-κB signaling on genome-wide transcription, we investigated the gene expression profile of primary fibroblasts isolated from wild-type and *p100^−/−^* mouse embryos [20]. We have chosen this experimental system since the lack of p100 in mice results in multiple defects that affect organization and function of lymphoid tissues [20]–[22], thereby exerting secondary effects on gene expression. In contrast to *Nfkb2^−/−^* mouse embryonic fibroblasts (MEFs), which are deficient in both p100 and p52 [15], *p100^−/−^* MEFs lack only the p100 inhibitor but still express the p52 subunit [20]. Passage 3 MEFs were left untreated and protein extracts and total RNA were isolated. Western blots were performed to verify increased levels of p52 in nuclei of *p100^−/−^* MEFs. We also observed higher nuclear RelB levels in *p100^−/−^* compared to wild-type MEFs, whereas RelA levels remained unchanged (Figure 1A). Furthermore, electrophoretic mobility shift assays revealed strongly increased κB DNA-binding activity in *p100^−/−^* vs. wild-type MEFs (Figure 1B). Dissection of κB DNA-binding protein complexes revealed that the majority contained p52 and RelB (Figure 1B). Of note, RelA binding was not activated in *p100^−/−^* compared to wild-type MEFs (Figure 1A and B).

To identify p52/RelB/p100-regulated genes we carried out microarray analysis using total RNA from the experiment described above hybridized to CodeLink Mouse Whole Genome Bioarrays. We identified 73 differentially expressed genes (minimum detectable fold change = 1.8; *P*<0.05) which are depicted as fold-change heatmap in Figure 1C. Gene expression changes were between +6-fold (up) and −20-fold (down). In the light of our recent gene expression profiling study [27], which identified 528 LTβR-responsive transcripts in mouse fibroblasts, the number of significantly regulated genes was surprisingly low. One possible explanation is that p52/p100 target genes are predominantly expressed in lymphoid tissues and that this is only partially recapitulated by MEFs. In the previous LTβR transcriptome study the majority of regulated genes (366) was dependent on both RelA and RelB. The markedly reduced number of genes in this experiment together with the nuclear translocation pattern (Figure 1A) and the EMSA results (Figure 1B) strongly suggest that RelA is not the main transcription factor driving constitutive gene expression in *p100^−/−^* MEFs. Table S1 shows a detailed list of regulated genes (probe, gene symbol, description, accession number, *P* values, and fold-change). Changes in mRNA levels of selected genes were confirmed by quantitative real-time reverse-transcription-PCR (qRT-PCR) from RNA isolates that were also used in microarray experiments (Figure 1C). Microarray results were largely confirmed by qRT-PCR analysis. From the 20 genes tested, 16 (*Ltc4s, Serpina3g, Traf1, Cd34, Rrad, Dclk1, Wnt10a, Enpp2/Atx, Ccl20, Igfbp4, Bmp4, Kazald1, Igfbp5, Mcpt8, Gdf6, Nfkb2*) were confirmed (Figure 1C and Table S2), and four (*Nod2, Ccl8, Fzd5, 6330577E15Rik*) could not be verified by qRT-PCR (Table S2).

**Figure 2:**
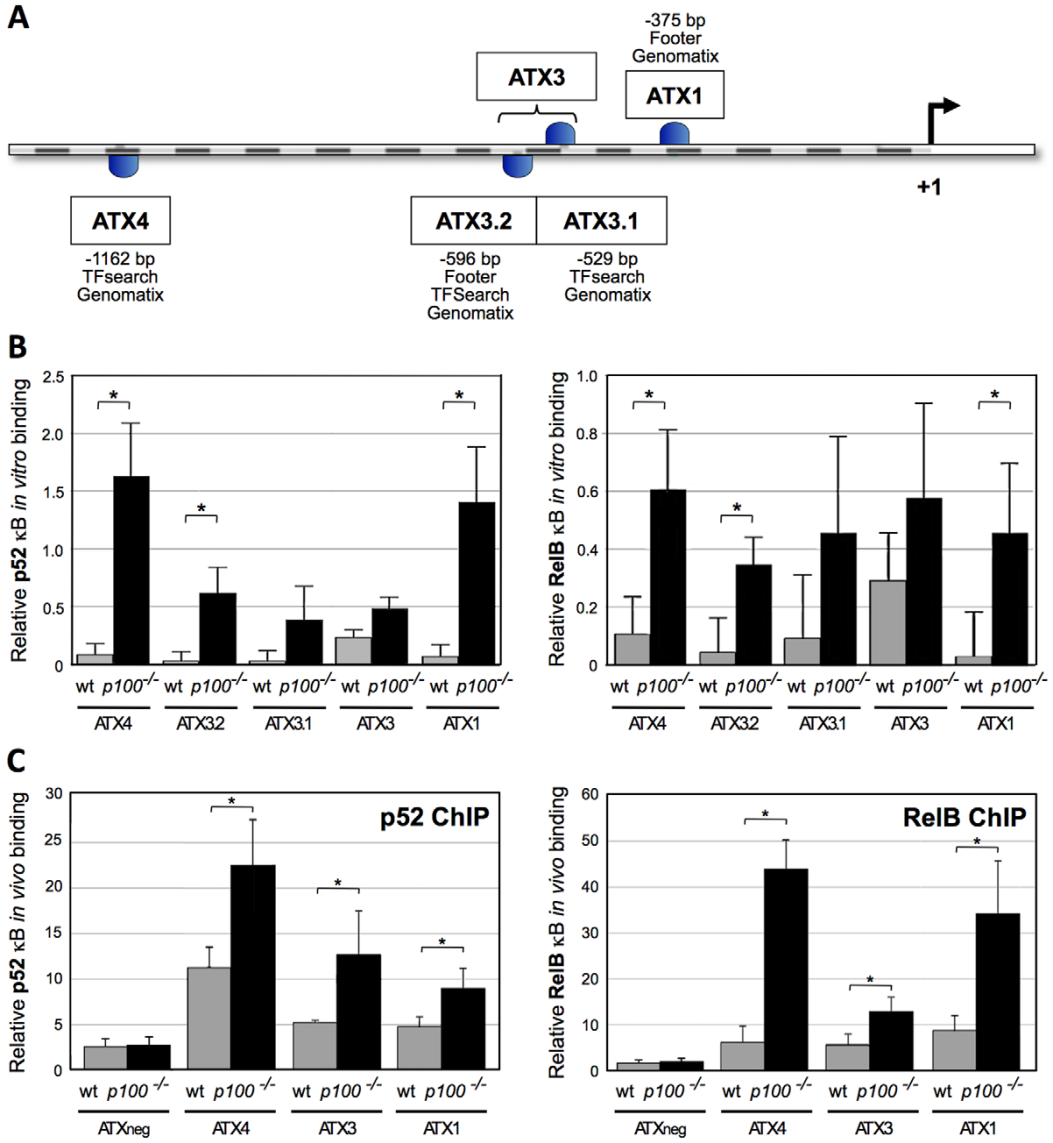
Lack of p100 results in enhanced p52 and RelB binding to NF-κB target sites in the *Enpp2/Atx* promoter. (A) Schematic view of the *Enpp2/Atx* promoter. *In silico* analysis of the *Enpp2/Atx* promoter (−2485 bp upstream of the ATG codon) with MatInspector and TFSearch softwares revealed four potential NF-κB sites: ATX4 at position −1162 (CGGGGGCTTC), ATX3.2 at position −596 (GGAAGCTCCC), ATX3.1 at position −529 (AGGGTCATTCC), and ATX1 at position −375 (GGGAAATTCT). ATX3.2 and ATX1 κB target site have been previously described using the software Footer [30]. (B) *In vitro* binding of p52 and RelB to κB target sites in the *Enpp2/Atx* promoter was determined by the TransAM Flexi NF-κB Family Transcription Factor Assay (Active Motif). Three independent experiments were performed. Data are presented as mean values ± SD. Statistically significant differences are indicated by * (Student's *t*-test, *P*≤0.05). (C) *In vivo* binding of p52 and RelB to κB target sites in the *Enpp2/Atx* promoter. An unrelated site (ATXneg) served as a negative control. For ChIP experiments, the Express Magnetic Chromatin Immunoprecipitation Kit (Active Motif) was employed according to the manufacturer's instructions. ChIP assays for RNA polymerase II binding to the *Gapdh* promoter were used for normalization as input control. Data are presented as mean values ± SD, n = 3. Statistically significant differences are indicated by * (Student's *t*-test, *P*≤0.05).

As the alternative NF-κB pathway is a major factor in controlling secondary lymphoid organ development and since *Enpp2* mRNA levels were increased in *p100^−/−^* vs. wild-type MEFs we investigated whether RelB and p52 directly regulate *Enpp2* mRNA expression. Enpp2 (also known as autotaxin, ATX) is a recently described molecule involved in lymphocyte homing [31]. In addition to NFAT1 and HOXA13, NF-κB/RelA has been suggested to regulate *Enpp2* expression in LPS-stimulated dendritic cells [32], [33], [34]. *In silico* analysis of the mouse *Enpp2/Atx* promoter identified four putative κB target sites: ATX4, ATX3.2, ATX3.1, and ATX1 (Figure 2A). *In vitro* assays showed that binding of p50 and RelA to any of the four κB sites was close to background and unaffected by the loss of p100 (Figure S1). In contrast, binding of both p52 and RelB to the putative κB sequences - in particular to sites ATX4, ATX3.2, and ATX1 - was strongly increased in *p100^−/−^* compared to wild-type MEFs. The κB sites ATX3.2 and ATX3.1 are separated by ca. 70 bp. However, we did not observe cooperative binding of RelB or p52 to these κB sequences (Figure 2B). Binding of NF-κB subunits to unrelated sequences (ATXunr1 and ATXunr2) in the *Enpp2/Atx* promoter was close to background and unchanged between wild-type and *p100^−/−^* MEFs (data not shown). Chromatin immunoprecipitation (ChIP) experiments verified direct binding of p52 and RelB to the ATX κB sites *in vivo* (Figure 2C). Thus, loss of p100 results in increased p52 and RelB binding to κB sites in the *Enpp2/Atx* promoter, indicating a direct transcriptional regulation of *Enpp2/Atx* by the alternative NF-κB pathway.

We next explored the consequences of constitutive alternative NF-κB signaling on genome-wide transcription in MEFs and examined on a global scale the biological themes that were influenced by alternative NF-κB activation. We started out with gene ontology (GO) enrichment analysis of significantly regulated genes to identify biological processes, molecular functions, and cellular components putatively regulated in *p100^−/−^* compared to wild-type MEFs. In the division of molecular function lack of p100 influenced only a tight branch of activities (focused on child terms of receptor binding/growth factor binding): “G-protein-coupled receptor binding” (GPCRB), “cytokine activity” (CA), and “insulin-like growth factor binding” (IGFB) (Figure 1C and D; Figure S2A). Within the division of cellular components, the p100 mutation influenced “extracellular region” (ER)-related themes whereas terms associated with intracellular regions were not significantly regulated (Figure 1C and D; Figure S2B). We observed the highest number of regulated terms in the division of biological processes (Figure 1C and D; Figure S2C, D, and E). In concert with our observations in the division of cellular components, the lack of p100 also affected the term “extracellular structure organization and biogenesis” (ESO). However, there was also a clear impact of the p100 mutation on development/morphogenesis (D/M) related issues, since “developmental processes” and its several specialized terms were significantly influenced. Interestingly, an unexpected branch of “anatomical structure development” was also affected by the deregulation of alternative NF-κB signaling: brain and nervous system development (B/ND) related processes like “forebrain development” and “nervous system development” were influenced by the mutation (Figure 1C and D; Figure S2C). Moreover, p100 also influenced issues related to cell growth/size (CG/S) (Figure 1C and D; Figure S2D). Furthermore, as expected from constitutive alternative NF-κB signaling “immune response/response to external stimulus” (IR/RES) related processes were also significantly regulated. In addition, topics related to migration/locomotion/taxis (M/L/T), which are intuitively targets of a deregulated alternative NF-κB pathway, were dysregulated in the absence of the p100 inhibitor. These were “behavior” and its child terms “locomotory behavior”, “taxis”, and “chemotaxis” (Figure 1C and D; Figure S2E).

**Figure 3:**
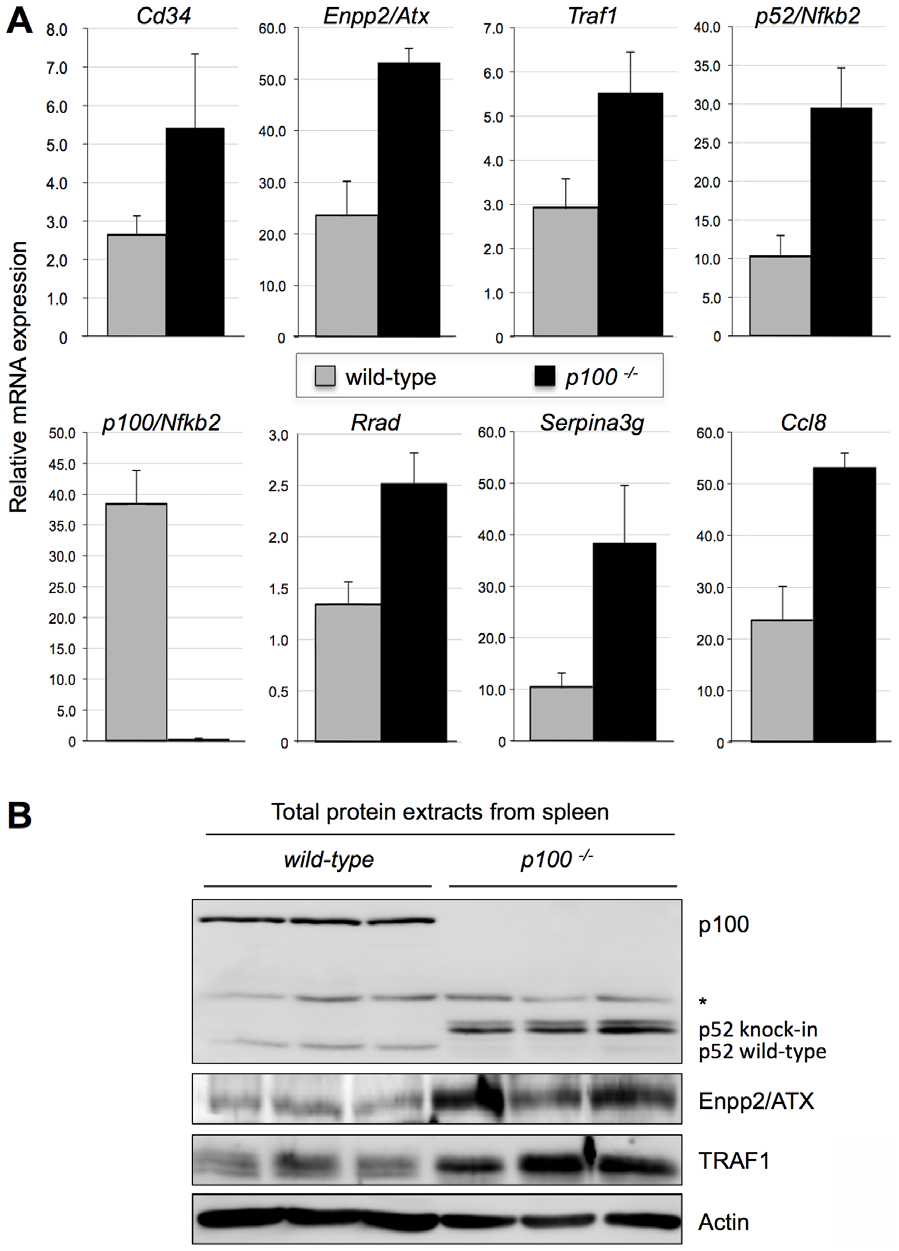
Gene expression analysis of wild-type and *p100^−/−^* spleens. (A) Changes in mRNA levels of selected genes were analyzed by qRT-PCR using RNA samples isolated from spleens of four wild-type and four *p100^−/−^* animals. Data are expressed as mean values ± SD. Differences were analyzed by Welch tests. *P*≤0.05 was considered significant. All genes shown were significantly differentially regulated in wild-type compared to mutant mice. (B) Western blot analysis of total protein extracts from wild-type and *p100^−/−^* spleens (30 µg/sample; protein extracts from three independent spleens are shown for each genotype) for the presence of p100/p52, ENPP2/ATX, and TRAF1. p52 wild-type protein and p52 protein resulting from the knock-in of the stop codon migrated with different speed due to a few amino acids difference in length. The asterisk indicates an unspecific signal. The membrane was probed for β-actin as a loading control.

Collectively, gene ontology analysis revealed enrichment of terms related to development/structure morphogenesis, extracellular region, but also to cell growth, inflammatory response, taxis/locomotory behavior, suggesting structural rearrangements in organs of *p100^−/−^* mice, which is in agreement with earlier studies [20], [21]. As the alternative NF-κB signaling pathway has a pivotal role in the development and maintenance of secondary lymphoid organs, we examined selected genes annotated to the above GO terms. Expression analysis of *p100^−/−^* vs. wild-type spleens showed significant upregulation of genes with anti-apoptotic/proliferative activity (*Enpp2/Atx*, *Serpina3g*, *Traf1*, *Rrad*), chemotactic/locomotory activity (*Enpp2/Atx*, *Ccl8*), and lymphocyte homing activity (*Enpp2/Atx*, *Cd34*). Gene expression of *Nfkb2*, as assayed with 3′-end primers, was diminished in *p100^−/−^* spleen as this mutant lacks the 3′-end of the gene. Expression of the remaining 5′-part, encoding p52, was increased since p52 exerts a positive feedback loop on its own regulation [20] (Figure 3A). ENPP2/ATX was also tested in Western blots. As expected, ENPP2/ATX protein levels were increased in spleen of *p100^−/−^* vs. wild-type mice. Expression of the anti-apoptotic protein TRAF1 was also elevated in spleens of *p100^−/−^* mutant mice (Figure 3B).

**Figure 4:**
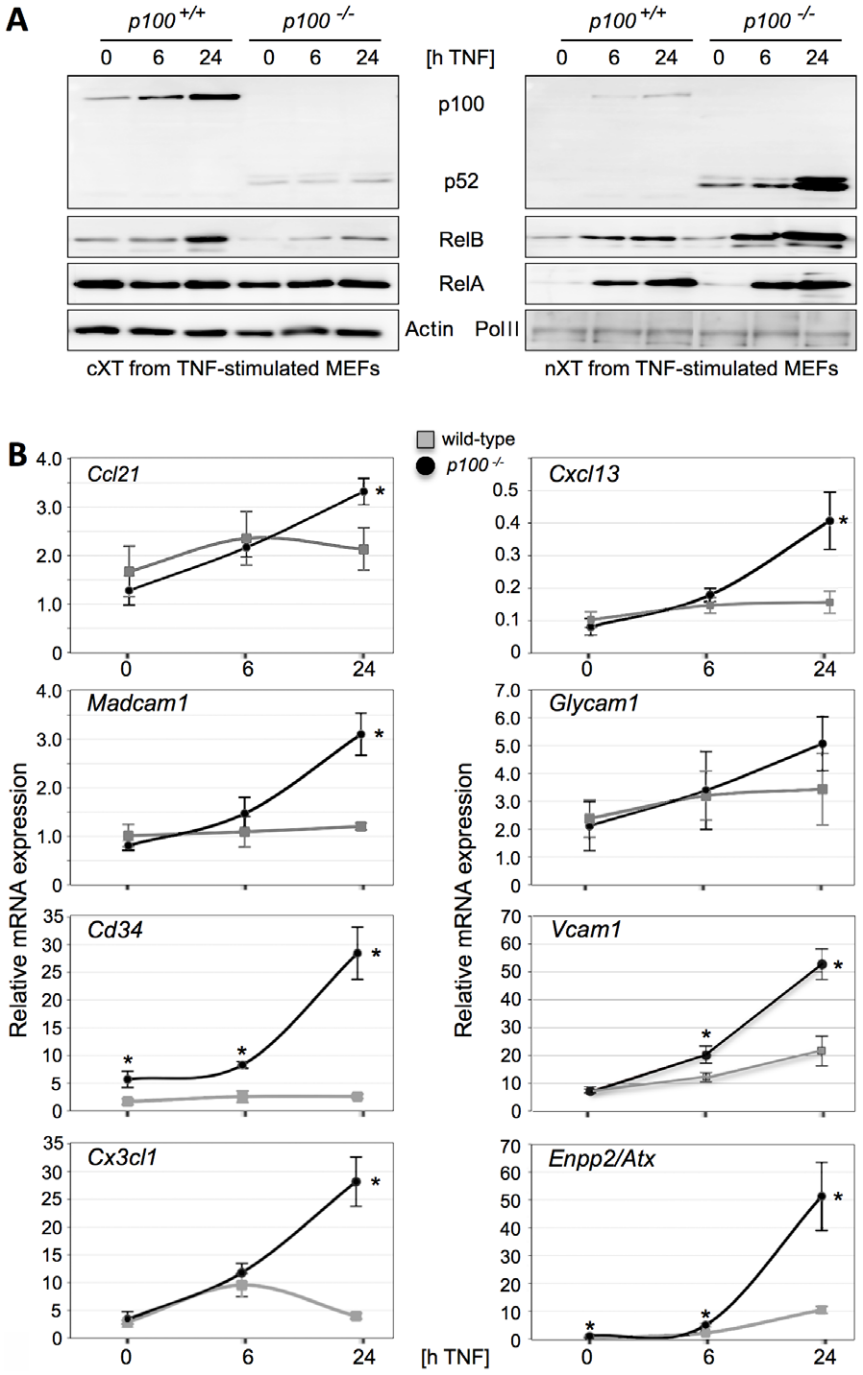
TNF synergizes with the lack of p100 in the induction of target gene expression. (A) Western blot of NF-κB family members in cytoplasmic and nuclear protein extracts (15 µg/sample) from unstimulated (0 h) and TNF-stimulated (6 and 24 h, 20 ng/ml recombinant murine TNF) wild-type and *p100^−/−^* MEFs. As cytoplasmic and nuclear loading controls β-actin and RNA Pol II were assayed, respectively. (B) Changes in mRNA levels of selected genes were analyzed by qRT-PCR. From 16 analyzed genes, 12 responded synergistically to TNF and the lack of p100 whereas only four genes responded similarly to TNF treatment of wild-type (grey squares) and *p100^−/−^* MEFs (black circles; see also Figure S4). qRT-PCR data represent n = 3 independent TNF stimulation experiments and are expressed as mean values ± SD. Differences between wild-type and *p100^−/−^* MEFs at each time-point were analyzed by Welch tests. *P*≤0.05 was considered significant (*).

In agreement with a previous report [21], we observed increased *Ccl21*, *Baff*, *Vcam1*, *Icam1*, *Madcam1*, and *Glycam1* mRNA expression in spleens of *p100^−/−^* mice compared to wild-type controls (data not shown). In addition, mutant spleens had increased mRNA expression of the proinflammatory chemokines encoding genes *Cx3cl1* and *Cxcl10* (Figure S3). Surprisingly, none of these genes was regulated in *p100^−/−^* vs. wild-type MEFs (data not shown). Therefore, we reasoned that in contrast to *p100^−/−^* MEFs kept under non-stimulating cell culture conditions, splenocytes of *p100^−/−^* mice may provide additional signals that sensitize alternative NF-κB signaling. To test whether signals from the classical pathway cross-talk to alternative NF-κB activation we chose TNF, a well-known and selective inducer of the classical pathway [16]. As shown in Figure 4A, TNF treatment resulted in increased cytoplasmic p100 in wild-type and increased nuclear p52 levels in *p100^−/−^* MEFs. Nuclear accumulation of p52 was restricted to *p100^−/−^* cells and accompanied by a marked increase in nuclear RelB. This result reflects enhanced nuclear translocation of p52-RelB heterodimers due to the TNF-induced increase of the cytoplasmic pools of p52 and RelB in combination with the lack of the p100 inhibitor [16] (data not shown). In contrast to p52 and RelB, TNF-induced nuclear translocation of RelA was similar in both wild-type and mutant MEFs. Collectively, these data show that in the absence of the p100 inhibitor TNF stimulates nuclear translocation of alternative p52-RelB complexes (Figure 4A).

We next searched for genes that were synergistically regulated by the classical (TNF) and the alternative (*p100^−/−^*) NF-κB pathway. We found that genes encoding (*i*) lymphorganogenic chemokines (CCL21, CXCL13), (*ii*) proinflammatory chemokines (CX3CL1, CXCL10), (*iii*) molecules regulating lymphocyte homing (ENPP2/ATX, CD34), (*iv*) serine protease inhibitor Serpina3g, (*v*) a lymphocyte specific enhancer element (PU-box) binding transcription factor SpiB, and (*vi*) cell adhesion molecules (VCAM-1, ICAM-1, and MAdCAM-1) belong to this group (Figure 4B and Figure S4). Interestingly, expression of the cell adhesion molecule GlyCAM-1 was only moderately induced by TNF in both wild-type and *p100^−/−^* MEFs, whereas it was dramatically upregulated in *p100^−/−^* spleen [21]. This observation suggests that induction of GlyCAM-1 expression may depend on the synergistic interaction between a so far unknown signal with the alternative NF-κB pathway.

**Figure 5:**
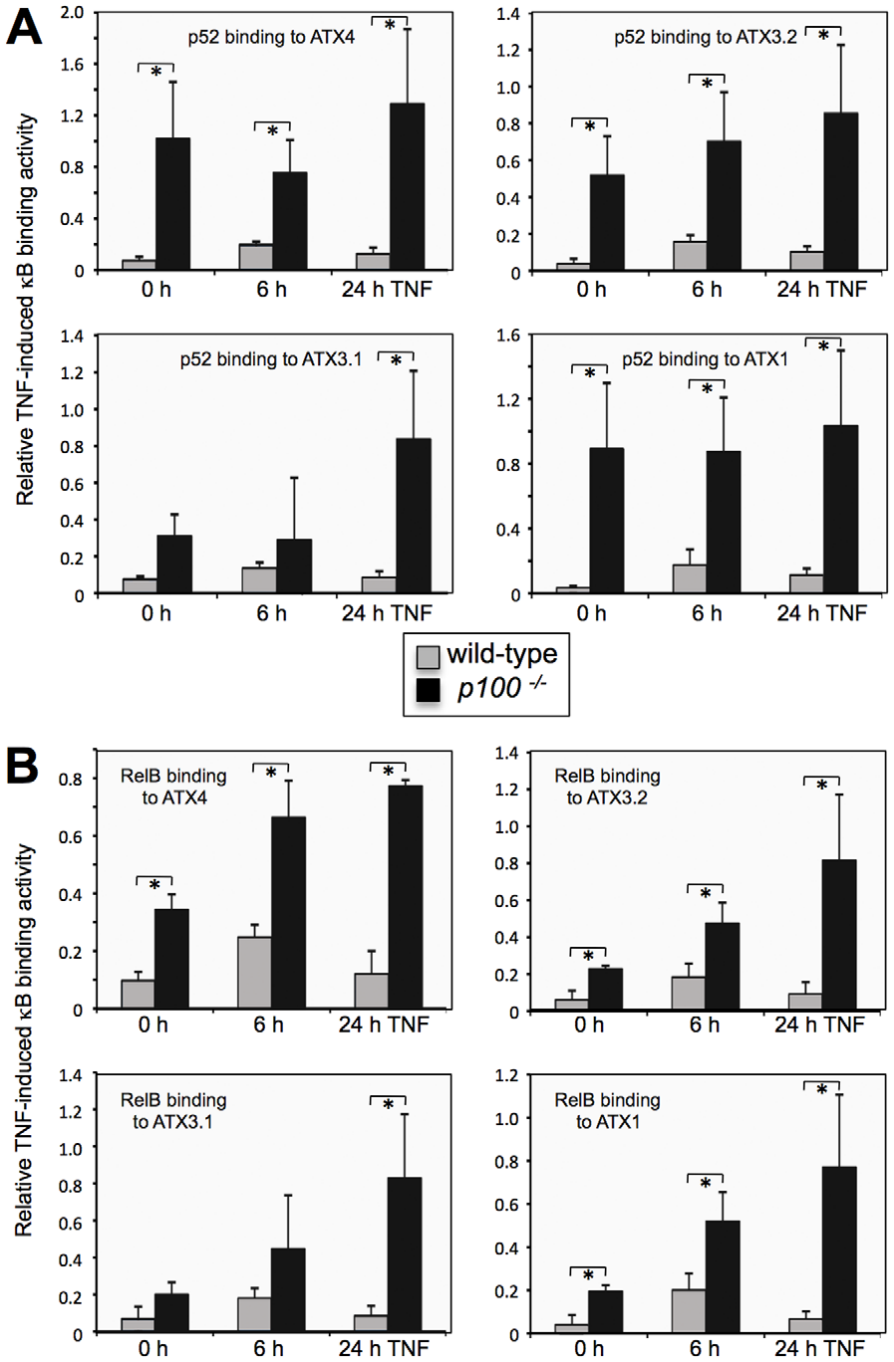
Lack of p100 cooperates with TNF to increase binding of p52 and RelB to the κB elements in the *Enpp2/Atx* promoter. Wild-type and *p100^−/−^* primary MEFs were stimulated with recombinant murine TNF (20 ng/ml) for 6 and 24 h or were left untreated and subsequently nuclear proteins were isolated. *In vitro* binding of NF-κB subunits p52 (A) and RelB (B) to the κB target sites in the *Enpp2/Atx* promoter was determined as in Figure 2. Data are presented as mean values ± SD from n = 3 independent experiments. Significant differences (*P*≤0.05) in p52 and RelB binding were calculated by Student's *t*-test and are indicated (*).

To investigate how the alternative NF-κB transcription factors ensure synergistic regulation of *Enpp2/Atx*, we assayed p52 and RelB binding to the ATX κB elements in response to TNF stimulation in wild-type and *p100^−/−^* MEFs. TNF treatment resulted in stronger DNA binding activities by both p52 and RelB in *p100^−/−^* compared to wild-type MEFs at each ATX κB site. This difference of *Enpp2/Atx* promoter binding was further increased upon prolonged TNF stimulation (Figure 5A and 5B), which is in agreement with the gene expression pattern of *Enpp2/Atx* in these cells (Figure 4B).

The second group of genes was regulated by TNF alone independent of the alternative NF-κB activation (Figure S4, right panels). These genes encoded predominantly proinflammatory chemokines such as CCL2, CCL7, CCL8, and CXCL1, which were also not differentially expressed under cell culture conditions *in vitro* (data not shown). Thus, whereas genes contributing to lymphocyte homing and lymphorganogenesis were synergistically regulated by the classical and alternative NF-κB pathways, most of the genes with inflammatory activity were regulated by the classical NF-κB pathway alone.

**Figure 6:**
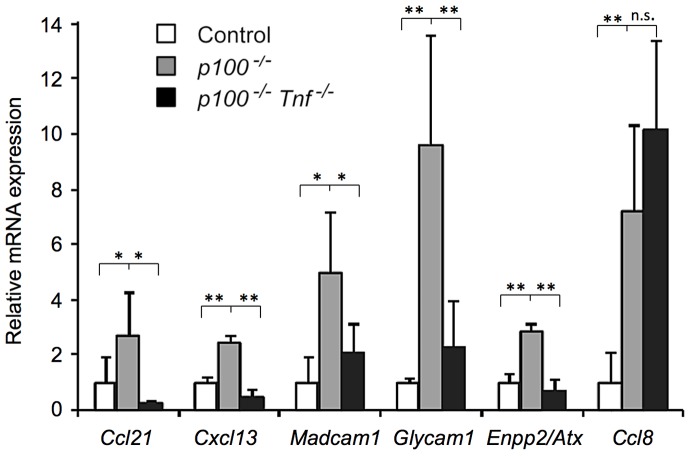
Synergistic regulation of gene expression by the classical (TNF) and the alternative (*p100^−/−^*) NF-κB pathway *in vivo*. Changes in mRNA levels of selected genes contributing to chemotaxis, lymphocyte homing, and cell adhesion were analyzed by qRT-PCR using RNA samples isolated from spleens of control (wild-type for the *Nfkb2* locus and heterozygous for the *TnfLta* locus), *p100^−/−^* (deficient for the *p100/Nfkb2* and wild-type for the *TnfLta* locus), and *p100^−/−^ Tnf^−/−^* (deficient for both the *p100/Nfkb2* and the *TnfLta* locus) animals. Four independent experiments were performed representing n = 6 control mice, n = 5 *p100^−/−^* mice, and n = 4 *p100^−/−^ Tnf^−/−^* mice. Data are presented as mean values ± SD. Significant differences (*P*≤0.05) were calculated by Student's *t*-test and are indicated (*). * *P*≤0.05; ** *P*≤0.01; n.s. = not significant.

To further investigate synergistic regulation exerted by TNF signaling in combination with the constitutively active alternative NF-κB pathway, we analyzed gene expression in spleens from control, *p100^−/−^*, and *p100^−/−^ TnfLta^−/−^* mutant animals. To allow a direct comparison with the data obtained from TNF-treated MEFs (see Figure 4), we focused our analysis on genes regulated by LTβR signaling in stromal cells [27], [28], [29], [30]. Expression of *Ccl21, Cxcl13, Madcam1, Glycam1, and Enpp2/Atx* was markedly upregulated in *p100^−/−^* compared to control spleen. This upregulation was strongly reduced in *p100^−/−^ TnfLta^−/−^* mutant spleen. In contrast, expression of *Ccl8* was not affected by TNF in *p100^−/−^* spleen, similar to the result from MEFs (Figure 6). Thus, the observed *in vitro* synergy between the lack of the p100 inhibitor and TNF signaling could largely be reproduced *in vivo*.

Several target genes of constitutive alternative NF-κB signaling have been involved in malignant processes: (*i*) TRAF1, an anti-apoptotic adaptor protein in TNF signaling, is upregulated in many lymphoid malignancies [35], [36], (*ii*) Rrad, a Ras related GTPase, promotes cell growth by accelerating cell cycle transitions [37], [38], and (*iii*) the lysophosphatidic acid producer Enpp2/ATX [39], [40]. Lysophosphatidic acid binds to its cognate G protein-coupled receptor through which it exerts several biological functions, such as increasing cell motility and stimulating cell proliferation, rendering Enpp2/ATX an important player in cell migration and homing [31]. In addition, enhanced expression of Enpp2/ATX has repeatedly been demonstrated in various malignant tumor tissues including non-small cell lung cancer [41], breast cancer [42], [43], renal cell cancer [44], hepatocellular carcinoma [45], thyroid cancer [46], and glioblastoma multiformae [47], suggesting that Enpp2/ATX confers tumorigenic and metastatic potential to cancer cells. Importantly, we could show that binding of p52 and RelB to the promoter of the *Enpp2/Atx* gene was induced in *p100^−/−^* MEFs. Moreover, we observed enhanced expression of anti-apoptotic/proliferative (*Enpp2/Atx*, *Serpina3g*, *Traf1*, *Rrad*), chemotactic/locomotory (*Enpp2/Atx*, *Ccl8*), and lymphocyte homing (*Enpp2/Atx*, *Cd34*) activities in spleens of *p100^−/−^* mice, suggesting that incorrect regulation of the alternative NF-κB pathway disturbs spleen homeostasis. Most importantly, biochemical and gene expression analyses of TNF-treated wild-type and *p100^−/−^* MEFs and gene expression analyses of mutant mice indicate a strong synergism between the classical and alternative NF-κB pathways, suggesting that unrestricted alternative NF-κB activation is further enhanced by classical NF-κB signaling *in vivo*. Thus, our data indicate that inflammatory cues such as TNF strongly synergize with alternative NF-κB signaling, resulting in an increased activation of alternative NF-κB target genes. Conversely, the status of p100 strongly influences the expression of TNF target genes/inflammatory genes.

## Materials and Methods

### Mice

Generation of *p100^−/−^* [20] and *TnfLta^−/−^* mice [48] has been described previously. All animals were housed and bred under standardized conditions with water and food *ad libitum* in a specific pathogen free mouse facility at the Leibniz-Institute for Age Research – Fritz-Lipmann-Institute, Jena. Animal care and experimental procedures were performed in accordance with accepted standards of animal welfare and approved by the Animal Care and Use Committee of the TLLV (Thüringer Landesamt für Lebensmittelsicherheit und Verbraucherschutz), Bad Langensalza, Germany. Approval IDs: J-SHK-2684-05-04-04-FLI/08 (Breeding and husbandry of mice), 22-2684-04-03-102/08 (Marking and genotyping of mice), and TöA/FLI-149-08 (Removal of organs for scientific purposes).

### Cell culture

Mouse embryonic fibroblasts (MEFs) were isolated from E14.5 wild-type and *p100^−/−^* mouse embryos. Five independent wild-type and *p100^−/−^* fibroblast cell lines each were cultured at 37°C in Dulbecco's modified Eagle's medium (GIBCO/Invitrogen, Karlsruhe, Germany) supplemented with 10% heat-inactivated fetal bovine serum (PAA Laboratories, Cölbe, Germany), penicillin (100 U/ml), streptomycin (100 µg/ml) and L-glutamine (2 mM) (GIBCO/Invitrogen). At passage 3, total RNA was isolated for microarray experiments. To monitor signaling events in parallel, cytoplasmic and nuclear proteins were extracted. For TNF induction, wild-type and *p100^−/−^* MEFs were left untreated or were stimulated with recombinant murine TNF (20 ng/ml; Sigma-Aldrich, T7539) for 6 and 24 h.

### Cytoplasmic and nuclear protein extracts, Western blots, EMSA, antibodies

Nuclear and cytoplasmic protein fractions from MEFs were prepared as described [49]. Equal loading of nuclear and cytoplasmic proteins (20 µg/sample) was checked in Western blots with antibodies (Abs) against Pol II (sc-899), β-actin (sc-1616) (Santa Cruz Biotechnology, Heidelberg, Germany), and S6 ribosomal protein (#2217) (Cell Signaling Technology, Frankfurt am Main, Germany). Western blot analysis using the Abs mentioned above and Abs specific for p100/p52 (#4882; Cell Signaling), RelB, and RelA (sc-226 and sc-372; Santa Cruz) were essentially performed as previously described [16]. For Western blot analysis of total protein extracts from wild-type and *p100^−/−^* spleens 30 µg protein/sample were used and the presence of ENPP2/ATX (Santa Cruz, sc-66813), p100/p52 (Cell Signaling, #4882), and TRAF1 (Santa Cruz, sc-66813) was assayed with specific antibodies. As loading control Western blots were probed for β-actin (Santa Cruz, sc-1616). EMSAs were carried out as described in [16], [49], [50]. For supershift experiments 1 µl from the following antibodies raised in Rodrigo Bravo's laboratory were used [50]: p.i., pre-immune serum; anti-RelA antibody (α-RelA), anti-RelB antibody (α-RelB), and anti-p52 antibody (α-p52).

### TransAM assay and chromatin immunoprecipitation

*In silico* analysis of the mouse *Enpp2/Atx* promoter was done using the software MatInspector (http://www.genomatix.de/en/index.html) and TFSearch (http://www.cbrc.jp/research/db/TFSEARCH.html). *In vitro* binding of NF-κB subunits to the κB target sites in the *Enpp2/Atx* promoter was tested with the TransAM Flexi NF-κB Family Transcription Factor Assay Kit (Active Motif, Cat. No. 43298) according to the manufacturer's instructions. Protein extracts from wild-type and *p100^−/−^* passage 3 MEFs were prepared by the Nuclear Extract Kit (Active Motif, Cat. No. 40010) and 5 µg of nuclear protein extracts per sample were used subsequently for the TransAM Assay. Binding of NF-κB subunits to unrelated sequences (ATXunr1 and ATXunr2) in the *Enpp2/Atx* promoter was close to background and unchanged between different treatment groups and was used for normalization of κB site binding by the NF-κB transcription factors. Normalization was carried out by extracting the OD_450_ values of unrelated sequences from the OD_450_ values of κB sites of corresponding treatment groups. To determine differences of NF-κB DNA binding between wild-type and mutant cells, 3 independent TransAM experiments were carried out. Data are expressed as mean ± standard deviation (SD). Differences were analyzed by Student's *t*-test. *P*≤0.05 or less was considered significant (*). Sequences of oligonucleotides containing NF-κB binding sites and unrelated control sites from the *Enpp2/Atx* promoter are listed in Table S4A.

*In vivo* binding of NF-κB subunits to the κB target sites and to an unrelated site (ATXneg, amplified by primer pairs ATXneg3) in the *Enpp2/Atx* promoter was tested by chromatin immunoprecipitation (ChIP) with the ChIP IT Express Magnetic Chromatin Immunoprecipitation Kit from Active Motif (Cat. No. 43298). ChIPs were carried out according to the manufacturer's instructions at 4°C for 4 h using 4 µg sheared chromatin and 3 µg Abs (RelB, NF-κB2 p52/p100, and RNA Pol II from Santa Cruz; sc-226, sc-298, and sc-899, respectively). As negative control rabbit IgG from Jackson ImmunoResearch (#011-000-003) was applied. ChIP assays of RNA Pol II binding to the *Gapdh* promoter were used for normalization. qPCR primers used for ChIP are listed in Table S4B.

### RNA isolation

Total cellular RNA was isolated using the RNeasy Mini Kit (Qiagen, Hilden, Germany) according to the manufacturer's instructions. Possible contamination by genomic DNA was removed by DNaseI treatment using the RNase-Free DNase Set (Qiagen). Quality of RNA samples was checked by spectrophotometry and agarose gel electrophoresis. RNA samples (2 µg total RNA per sample) were used for cRNA preparation for microarrays only when the ratio A260:A280 was 1.8–2.1 and the RNA was intact.

### Microarrays

Microarray analysis was performed using CodeLink Mouse Whole Genome Bioarrays containing 36227 probes (GE Healthcare, Munich, Germany). This one-color system harbors for each of the investigated 34967 transcripts one 30mer oligo probe spotted per slide. For gene expression profiling, 5 independent cell lines of each wild-type and *p100^−/−^* MEFs were used. Passage 3 MEFs were left untreated and total RNA was isolated as described above followed by cRNA synthesis. cRNA from each treatment group was hybridized onto distinct bioarrays, resulting in 5 wild-type and 5 *p100^−/−^* bioarray hybridizations. cRNA target preparation, bioarray hybridization and detection was carried out according to the manufacturer's protocol provided with the CodeLink iExpress Assay Reagent Kit (GE Healthcare). For scanning microarrays, a GenePix 4000B Array Scanner and GenePix Pro 4.0 software (Axon Instruments Inc./Molecular Devices, Munich, Germany) were employed according to settings suggested by the supplier of CodeLink bioarrays (GE Healthcare) in the protocol provided with the CodeLink iExpress Assay Reagent Kit (GE Healthcare). Microarray data are MIAME compliant and raw data have been deposited in NCBIs database GEO (http://www.ncbi.nlm.nih.gov/geo/) as detailed on the MGED Society website (http://www.mged.org/Workgroups/MIAME/miame.html. Data are accessible through the GEO series accession number GSE30317.

### Microarray data preprocessing

Microarray raw data of wild-type and *p100^−/−^* MEFs were analyzed using the Codelink™ Expression Analysis v4.1 software (GE Healthcare) and MDFC values were extracted. All subsequent analyses were performed using R and Bioconductor. For the analysis only genes with probe type ‘DISCOVERY’ were considered (34967 genes). To remove negative expression values (local background>spot intensity) raw intensities with values <0.01 were set to 0.01. The raw intensities of each array were scaled to the array median. After logarithmizing the expression values, quantile normalization was applied across all arrays.

### Differentially expressed genes

A gene was included in the data analysis if it was flagged ‘G’ (good) or ‘S’ (contains saturated pixels) on at least two arrays in any of the two groups (wild-type or *p100^−/−^*). Furthermore, genes selected were required to have a FC higher than or equal to the FC threshold determined from the maximum MDFC (1.8) in these groups. To identify genes significantly differentially expressed after stimulation, a Student's *t*-test was performed for the previously filtered genes. The resulting *P* values were corrected for multiple testing using the method of Benjamini and Hochberg. Allowing a false discovery rate of 5%, a total of 73 genes were identified that were significantly regulated in *p100^−/−^* versus wild-type cells.

### Functional analysis with GO

Analysis of functional enrichment was performed employing Fisher's exact test. The resulting *P* values (*P*<0.01) were used to rank GO terms according to their significance. Terms with less than 3 genes on the list of investigated genes were regarded as too specific, and excluded from the analysis. Expert knowledge was used to assign broader themes to specific GO categories.

### qRT-PCR

For qRT-PCR, first strand cDNA was obtained from 2 µg of total RNA for each treatment group using oligo-dT primers and M-MLV Reverse Transcriptase kit (Promega, Mannheim, Germany) according to manufacturer's protocols. qRT-PCRs were performed in an iCycler Thermal Cycler real-time PCR machine (Bio-Rad Laboratories, Hercules, CA) using SYBR Green I as detector dye and reagents from the Quantace SensiMix DNA Kit (Quantace Ltd., Watford, UK). Primers for qRT-PCRs with Tm of 60°C were designed using Primer3 software (v. 0.4.0; http://frodo.wi.mit.edu/primer3/) [51]. For normalization, *β-actin* was used as an endogenous reference gene to correct for variation in RNA content and variation in the efficiency of the reverse transcription reaction. Statistical analysis of qRT-PCR results was made from (*i*) 5 independent biological samples per genotype for confirmation of the microarray results (*ii*) 3 independent TNF stimulation experiments, and (*iii*) 4 spleens per genotype to investigate gene expression patterns from spleen. Data are expressed as mean values ± SD. Differences were analyzed by Welch test. *P* values of 0.05 or less were considered significant (*). Sequences of forward and reverse primers are shown in Table S3.

## Supporting Information

Figure S1 **Binding of p50 and RelA to any of the four κB sites was not affected by the loss of p100.** *In vitro* binding of the NF-κB subunits p50 (A) and RelA (B) to the putative κB target sites in the *Enpp2/Atx* promoter was determined by the TransAM Flexi NF-κB Family Transcription Factor Assay. Negative values result from subtracting unspecific binding of p50 or RelA to DNA sequences unrelated to κB sites (ATXunr). To determine differences of NF-κB DNA binding between wild-type and mutant cells, three independent TransAM experiments were carried out. Data are expressed as mean values ± SD. Differences were analyzed by Student's *t*-test. Binding differences between nuclear extracts from wild-type and *p100* ^−/−^ MEFs did not reach significance (*P*>0.05).
(TIF)

Figure S2 **Gene Ontology analysis of significantly differentially regulated genes.** (A) Division of molecular function. Lack of p100 influenced: “G-protein-coupled receptor binding” (GPCRB), “cytokine activity” (CA), and “insulin-like growth factor binding” (IGFB). The single terms and their GO number significantly differentially regulated between wild-type and *p100^−/−^* MEFs are shown in blue. (B) Division of cellular components. The p100 mutation influenced “extracellular region” (ER)-related themes. The single terms (and their GO number) significantly regulated are shown in blue. (C, D, and E) Division of biological processes. Three main branches proved to be influenced by the lack of the p100 molecule. (C) Biological process 1. The p100 mutation affected “extracellular structure organization and biogenesis” (ESO), “development/morphogenesis (D/M) related issues”, and “brain and nervous system development” (B/ND) related processes. The specialized terms (and their GO numbers) influenced by the mutation are shown in blue. (D) Biological process 2. p100 deficiency influenced issues related to “cell growth/size” (CG/S). The single terms (and their GO number) significantly regulated are shown in blue. (E) Biological process 3. The absence of the p100 inhibitor influenced “immune response/response to external stimulus” (IR/RES) and “migration/locomotion/taxis” (M/L/T). The specialized terms (and their GO numbers) significantly regulated in the mutant are shown in blue.
(TIF)

Figure S3 ***p100^−/−^*** **spleens displayed increased mRNA expression of the proinflammatory chemokines genes** ***Cx3cl1*** **and** ***Cxcl10*** **.** Changes in mRNA levels of these two genes were analyzed by qRT-PCR using RNA samples isolated from spleens of wild-type and *p100^−/−^* animals (n = 4 each). Data are expressed as mean values ± SD. Differences were analyzed by Welch tests. Both genes were significantly upregulated (*P*≤0.05) in *p100^−/−^* versus wild-type spleen (*).
(TIF)

Figure S4 **TNF synergized with the lack of p100 in the induction of target gene mRNA expression.** Wild-type and *p100^−/−^* MEFs were stimulated for 6 and 24 h with 20 ng/ml TNF or were left untreated. Changes in mRNA levels of selected genes were analyzed by qRT-PCR. Four additional genes that responded synergistically to TNF and the constitutive activation of the alternative NF-κB pathway are shown on the left (see also Figure 4B). Right panels depict four genes that did not show cooperative regulation by TNF and p100 deletion. Statistical significance of qRT-PCR results was calculated from n = 3 independent TNF stimulation experiments. Data are expressed as mean values ± SD. Differences between wild-type and *p100^−/−^* MEFs at each time-point were analyzed by Welch tests. *P*≤0.05 was considered significant (*).
(TIF)

Table S1 **Detailed list of genes differentially regulated in** ***p100^−/−^*** **versus wild-type MEFs as determined by microarray analysis.** Probe, gene symbol, description, accession number, *P* values, and fold-change and are shown.
(XLS)

Table S2 **Confirmation of microarray results by qRT-PCR analysis.** Gene symbol, fold-change of gene regulation in *p100^−/−^* over wild-type cells as assayed by microarray, *t*-test *P* values of microarray results, fold-change of gene regulation in *p100^−/−^* over wild-type cells as assayed by qRT-PCR, and *t*-test *P* value of qRT-PCR results are shown. Sixteen out of 20 genes could be verified by qRT-PCR, while four (*Nod2*, *Ccl8*, *Fzd5* and *6330577E15Rik*) could not be confirmed by this independent method.
(DOC)

Table S3 **List of primers used in qRT-PCR experiments.** Gene symbol and sequences of forward and reverse primers in 5′ to 3′ orientation are shown.
(DOC)

Table S4 **List of oligos and primers used in DNA binding assays.** (A) Sequences of oligos used in TransAM assays are shown in 5′ to 3′ orientation. Upper strand oligos were biotinylated at the 5′ end. Potential NF-κB binding sites are underlined. Lower strand oligos were left unmodified. (B) Sequences of qPCR primers used in ChIP are shown in 5′ to 3′ orientation.
(DOC)
