# Supplementary figures and images for: p100 Deficiency Is Insufficient for Full Activation of the Alternative NF-κB Pathway: TNF Cooperates with p52-RelB in Target Gene Transcription
Source: PLoS One. 2012 Aug 6;7(8):e42741. doi: 10.1371/journal.pone.0042741 (PMC3412832; doi:10.1371/journal.pone.0042741)

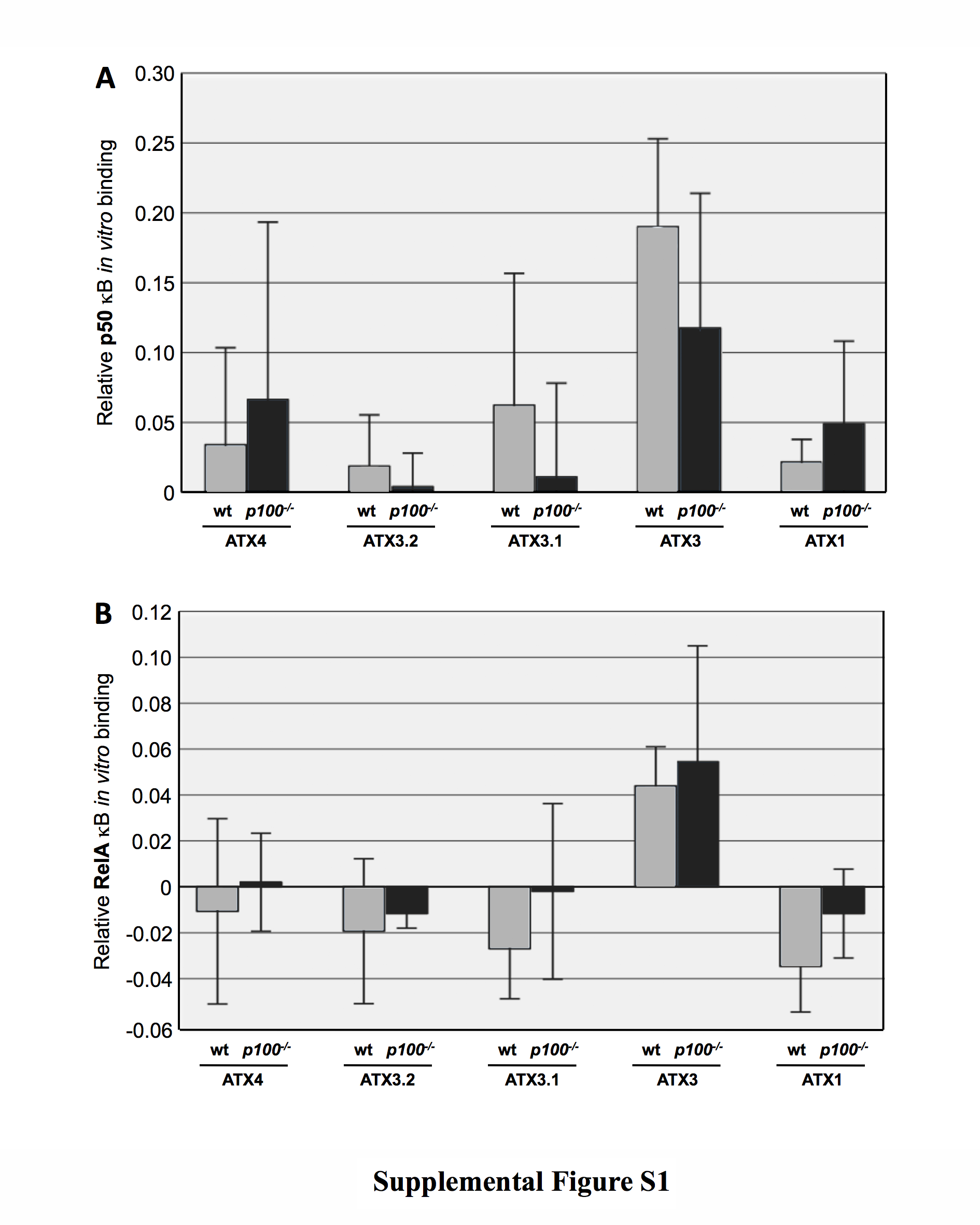

Supplement: Figure S1 — Binding of p50 and RelA to any of the four κB sites was not affected by the loss of p100. In vitro binding of the NF-κB subunits p50 (A) and RelA (B) to the putative κB target sites in the Enpp2/Atx promoter was determined by the TransAM Flexi NF-κB Family Transcription Factor Assay. Negative values result from subtracting unspecific binding of p50 or RelA to DNA sequences unrelated to κB sites (ATXunr). To determine differences of NF-κB DNA binding between wild-type and mutant cells, three independent TransAM experiments were carried out. Data are expressed as mean values ± SD. Differences were analyzed by Student's t-test. Binding differences between nuclear extracts from wild-type and p100 −/− MEFs did not reach significance (P>0.05). (TIF) [file pone.0042741.s001.tif]

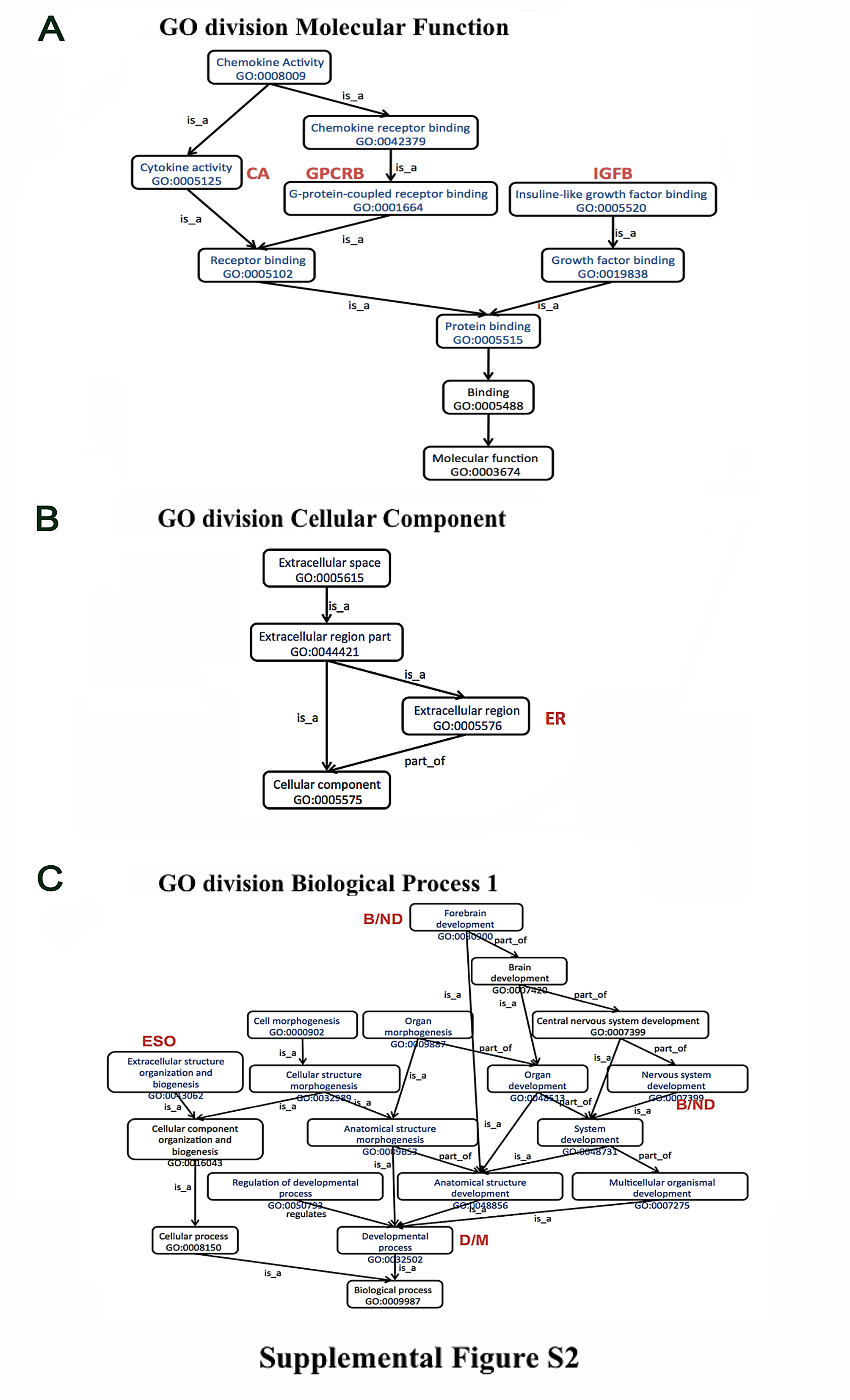

Supplement: Figure S2 — Gene Ontology analysis of significantly differentially regulated genes. (A) Division of molecular function. Lack of p100 influenced: “G-protein-coupled receptor binding” (GPCRB), “cytokine activity” (CA), and “insulin-like growth factor binding” (IGFB). The single terms and their GO number significantly differentially regulated between wild-type and p100−/− MEFs are shown in blue. (B) Division of cellular components. The p100 mutation influenced “extracellular region” (ER)-related themes. The single terms (and their GO number) significantly regulated are shown in blue. (C, D, and E) Division of biological processes. Three main branches proved to be influenced by the lack of the p100 molecule. (C) Biological process 1. The p100 mutation affected “extracellular structure organization and biogenesis” (ESO), “development/morphogenesis (D/M) related issues”, and “brain and nervous system development” (B/ND) related processes. The specialized terms (and their GO numbers) influenced by the mutation are shown in blue. (D) Biological process 2. p100 deficiency influenced issues related to “cell growth/size” (CG/S). The single terms (and their GO number) significantly regulated are shown in blue. (E) Biological process 3. The absence of the p100 inhibitor influenced “immune response/response to external stimulus” (IR/RES) and “migration/locomotion/taxis” (M/L/T). The specialized terms (and their GO numbers) significantly regulated in the mutant are shown in blue. (TIF) [file pone.0042741.s002.tif]

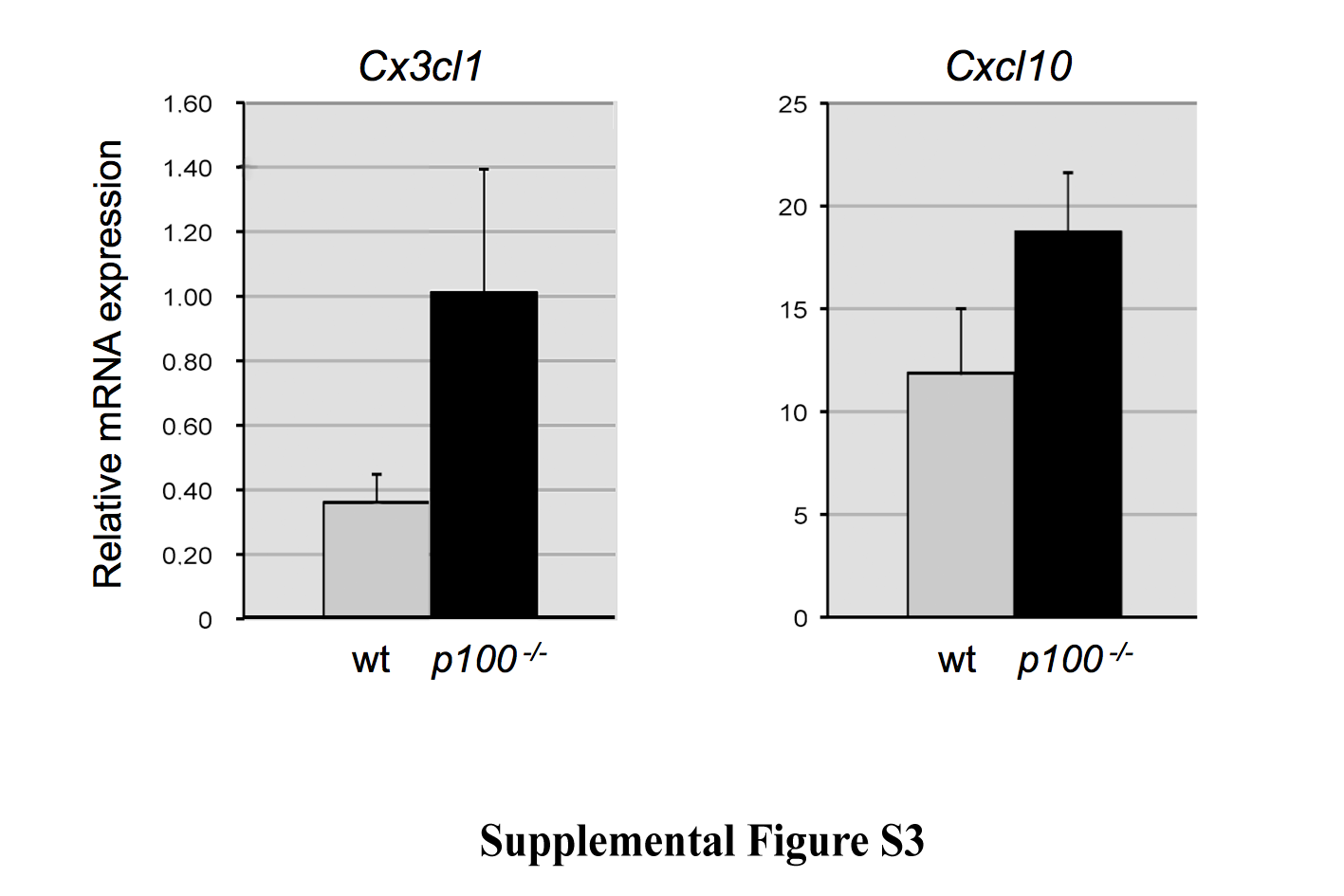

Supplement: Figure S3 — p100−/− spleens displayed increased mRNA expression of the proinflammatory chemokines genes Cx3cl1 and Cxcl10 . Changes in mRNA levels of these two genes were analyzed by qRT-PCR using RNA samples isolated from spleens of wild-type and p100−/− animals (n = 4 each). Data are expressed as mean values ± SD. Differences were analyzed by Welch tests. Both genes were significantly upregulated (P≤0.05) in p100−/− versus wild-type spleen (*). (TIF) [file pone.0042741.s003.tif]

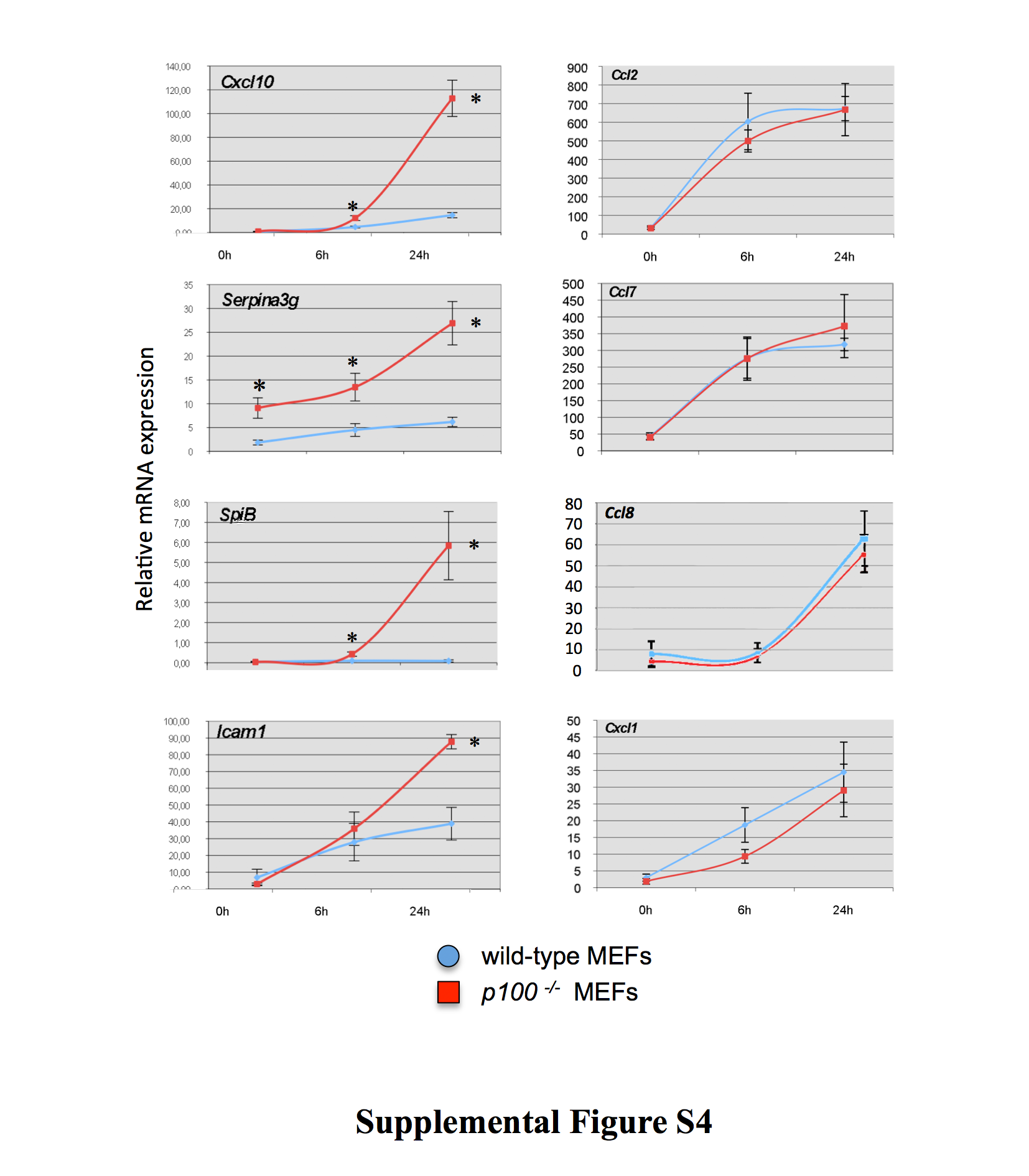

Supplement: Figure S4 — TNF synergized with the lack of p100 in the induction of target gene mRNA expression. Wild-type and p100−/− MEFs were stimulated for 6 and 24 h with 20 ng/ml TNF or were left untreated. Changes in mRNA levels of selected genes were analyzed by qRT-PCR. Four additional genes that responded synergistically to TNF and the constitutive activation of the alternative NF-κB pathway are shown on the left (see also Figure 4B). Right panels depict four genes that did not show cooperative regulation by TNF and p100 deletion. Statistical significance of qRT-PCR results was calculated from n = 3 independent TNF stimulation experiments. Data are expressed as mean values ± SD. Differences between wild-type and p100−/− MEFs at each time-point were analyzed by Welch tests. P≤0.05 was considered significant (*). (TIF) [file pone.0042741.s004.tif]
