# Supplementary material for: p100 Deficiency Is Insufficient for Full Activation of the Alternative NF-κB Pathway: TNF Cooperates with p52-RelB in Target Gene Transcription
Source: PLoS One. 2012 Aug 6;7(8):e42741. doi: 10.1371/journal.pone.0042741 (PMC3412832; doi:10.1371/journal.pone.0042741)
Supplement: Table S2 — Confirmation of microarray results by qRT-PCR analysis. Gene symbol, fold-change of gene regulation in p100−/− over wild-type cells as assayed by microarray, t-test P values of microarray results, fold-change of gene regulation in p100−/− over wild-type cells as assayed by qRT-PCR, and t-test P value of qRT-PCR results are shown. Sixteen out of 20 genes could be verified by qRT-PCR, while four (Nod2, Ccl8, Fzd5 and 6330577E15Rik) could not be confirmed by this independent method. (DOC) [file pone.0042741.s006.doc]

**Supplemental Table S2 – Verification of microarray results by qRT-PCR in MEFs**

| **Gene symbol** | **Fold-change (*p100^-/-^* vs. wild-type) Microarrays** | **t-test**  ***P* value Microarrays** | **Fold-change (*p100^-/-^* vs. wild-type) qRT-PCR** | **t-test**  ***P* value qRT-PCR** | **Remark** |
| --- | --- | --- | --- | --- | --- |
| ***Ltc4s*** | +6.0 | 0.00015 | +6.56 | 0.00634 | Verified |
| ***Serpina3g*** | +4.5 | 1e-04 | +6.28 | 0.00642 | Verified |
| ***Traf1*** | +3.0 | 0.00015 | +5.17 | 0.00020 | Verified |
| ***Cd34*** | +2.7 | 4.8e-05 | +2.87 | 0.00103 | Verified |
| ***Rrad*** | +2.6 | 8.5e-07 | +3.62 | 0.00290 | Verified |
| ***Dclk1*** | +2.4 | 0.00015 | +1.70 | 0.00179 | Verified |
| ***Wnt10a*** | +2.1 | 0.0044 | +2.40 | 0.04092 | Verified |
| ***Nod2*** | +2.0 | 0.028 | -1.02 | 0.91043 | Not verified |
| ***Ccl8*** | +1.9 | 0.039 | +1.38 | 0.29986 | Not verified |
| ***Enpp2/Atx*** | +1.9 | 0.041 | +2.26 | 0.00604 | Verified |
| ***Ccl20*** | -1.80 | 0.017 | -3.73 | 0.00169 | Verified |
| ***Igfbp4*** | -1.9 | 0.00093 | -2.27 | 0.00596 | Verified |
| ***Fzd5*** | -2.0 | 0.028 | -1.59 | 0.08625 | Not verified |
| ***Bmp4*** | -2.2 | 0.00028 | -2.73 | 0.00760 | Verified |
| ***Kazald1*** | -2.6 | 0.00028 | -3.54 | 0.00875 | Verified |
| ***Igfbp5*** | -2.6 | 0.026 | -1.95 | 0.02620 | Verified |
| ***Mcpt8*** | -3.5 | 0.00015 | -5.85 | 0.00076 | Verified |
| ***Gdf6*** | -4.6 | 0.018 | -4.30 | 0.02074 | Verified |
| ***Nfkb2*** | -14.0 | 0.00022 | -60.14 | 4.8e-05 | Verified |
| ***6330577E15Rik*** | -20.0 | 1.4e-07 | -1.04 | 0.75682 | Not verified |
