# Supplementary material for: p100 Deficiency Is Insufficient for Full Activation of the Alternative NF-κB Pathway: TNF Cooperates with p52-RelB in Target Gene Transcription
Source: PLoS One. 2012 Aug 6;7(8):e42741. doi: 10.1371/journal.pone.0042741 (PMC3412832; doi:10.1371/journal.pone.0042741)
Supplement: Table S3 — List of primers used in qRT-PCR experiments. Gene symbol and sequences of forward and reverse primers in 5′ to 3′ orientation are shown. (DOC) [file pone.0042741.s007.doc]

**Supplemental Table S3 – qRT-PCR primers**

| **Gene symbol** | **Forward qRT-PCR primer sequence (in 5’ to 3’ orientation)** | **Reverse qRT-PCR primer sequence (in 5’ to 3’ orientation)** |
| --- | --- | --- |
| ***Cx3cl1*** | ggctaagcctcagagcattg | cattttcctctggggttga |
| ***Enpp2/Atx*** | tggcttacgtgacattgagg | gtcggtgaggaaggatgaaa |
| ***Cxcl10/Ip10*** | aagtgctgccgtcattttct | gtggcaatgatctcaacacg |
| ***Nfkb2 5’ -end*** | gaagatctcccgaatggaca | cgtctgtgggagagaagtcc |
| ***Nfkb2 3’-end*** | GCTAATGTGAATGCCCGGAC | CTTTGGGTATCCCTCTCAGGC |
| ***Ccl7/Mcp3*** | AATGCATCCACATGCTGCTA | ATAGCCTCCTCGACCCACTT |
| ***Cxcl1/Kc*** | GCTGGGATTCACCTCAAGAA | TGGGGACACCTTTTAGCATC |
| ***Dclk1*** | cagatcttgatgggccaagt | catcattaacccagggatgc |
| ***Rrad*** | aagacggacctgaagcagaa | tttctcaaagctgcccttgt |
| ***Ltc4s*** | gcgagtactttccgctgttc | ctcgcgtataggggagtcag |
| ***Serpina3g*** | tggtctccagcaacactgac | ggggtctctgtgaggttgaa |
| ***Mcpt8*** | aaacacccagctcattcctg | ccaatccttgctctttggaa |
| ***Gdf6*** | agcctctgcacgtgaatttt | catcagcgtctgaatgatgg |
| ***Traf1*** | TGTGTGGCCGGACTGTCA | GCGCAGGCACAACTTGTAAC |
| ***Cd34*** | gcattggtcacctctggagt | attggcctttccctgagtct |
| ***β-actin*** | TGGCGCTTTTGACTCAGGA | GGGAGGGTGAGGGACTTCC |
| ***Dclk1*** | cagatcttgatgggccaagt | catcattaacccagggatgc |
| ***Ccl20*** | cgactgttgcctctcgtaca | aggaggttcacagccctttt |
| ***Wnt10a*** | catgagtgccagcatcagtt | aaccgcaagccttcagttta |
| ***Igfbp4*** | acagagccgtacccacga | aggaagcttcacccctgtct |
| ***Fzd5*** | tcttgtctgcgtgctacctg | ggccatgccaaagaaataga |
| ***Bmp4*** | tgatacctgagaccgggaag | agccggtaaagatccctcat |
| ***Kazald1*** | ggctggctacagattcaagc | cctcctcctcctcaggaaac |
| ***Igfbp5*** | aagcttccctccaggagttc | acttgtccacacaccagcag |
| ***Nod2*** | ctgtccaacaatggcatcac | agagtccaggcccctacagt |
| ***Ccl8*** | CAGACCAAGCAGGGTATG | TGCCTGGAGAAGATTAGGG |
| ***6330577E15Rik*** | atctaatgccctcccaaagg | gctgaccacacttcctccac |
