# Supplementary material for: p100 Deficiency Is Insufficient for Full Activation of the Alternative NF-κB Pathway: TNF Cooperates with p52-RelB in Target Gene Transcription
Source: PLoS One. 2012 Aug 6;7(8):e42741. doi: 10.1371/journal.pone.0042741 (PMC3412832; doi:10.1371/journal.pone.0042741)
Supplement: Table S4 — List of oligos and primers used in DNA binding assays. (A) Sequences of oligos used in TransAM assays are shown in 5′ to 3′ orientation. Upper strand oligos were biotinylated at the 5′ end. Potential NF-κB binding sites are underlined. Lower strand oligos were left unmodified. (B) Sequences of qPCR primers used in ChIP are shown in 5′ to 3′ orientation. (DOC) [file pone.0042741.s008.doc]

**Supplemental Table S4 – Oligos and primers used in DNA binding assays**

**A) TransAM Oligos (potential NF-κB binding sites are underlined)**

| **Oligo** | **Upper strand TransAM oligo sequence (in 5’ to 3’ orientation, biotinylated on 5’ end)** | **Lower strand TransAM oligo sequence (in 5’ to 3’ orientation, not modified)** |
| --- | --- | --- |
| **ATXunr1** | CTTCCAAGGAGAGCCTAGGTTTTCTTAGAGATGGGTTTCTATGTCAGAGA | TCTCTGACATAGAAACCCATCTCTAAGAAAACCTAGGCTCTCCTTGGAAG |
| **ATXunr2** | TGTTTTACAGTTGATTCGATGCCAAGATAAGGCTATCCTCCTGACTGCCT | AGGCAGTCAGGAGGATAGCCTTATCTTGGCATCGAATCAACTGTAAAACA |
| **ATX4** | GTGCTATCGGGTTTTATGCGAGCAGAGCCACGGGGGCTTCCTTTAAGCAG | CTGCTTAAAGGAAGCCCCCGTGGCTCTGCTCGCATAAAACCCGATAGCAC |
| **ATX3.2** | AATGTTATCTCTCGTGTGAGTTGCAATTTTGGAAGCTCCCATTGTGTGAA | TTCACACAATGGGAGCTTCCAAAATTGCAACTCACACGAGAGATAACATT |
| **ATX3.1** | AGTAGCTGACAATGTATTTATATAGATACAGGGTCATTCCAACTGCCAGA | TCTGGCAGTTGGAATGACCCTGTATCTATATAAATACATTGTCAGCTACT |
| **ATX3** | AATAATGTTATCTCTCGTGTGAGTTGCAATTTTGGAAGCTCCCATTGTGTGAAGCTCCCAAGGAATCAGAAGTAGCTGACAATGTATTTATATAGATACAGGGTCATTCCAACTGCCAGA | TCTGGCAGTTGGAATGACCCTGTATCTATATAAATACATTGTCAGCTACTTCTGATTCCTTGGGAGCTTCACACAATGGGAGCTTCCAAAATTGCAACTCACACGAGAGATAACATTATT |
| **ATX1** | ATAACTACATAGTAAACGCTTCGAGCTGATGGGAAATTCTGGGCAGCTCT | AGAGCTGCCCAGAATTTCCCATCAGCTCGAAGCGTTTACTATGTAGTTAT |

**B) ChIP qPCR primers**

| **Primer** | **Forward ChIP qPCR primer sequence (in 5’ to 3’ orientation)** | **Reverse ChIP qPCR primer sequence (in 5’ to 3’ orientation)** |
| --- | --- | --- |
| GAPDH1 | TGTACGGGTCTAGGGATGCT | GGCTGCAGGAGAAGAAAATG |
| ATXneg3 | CAACTTGCTCTCACGCTTTG | CAGCCTGTAGGTCCCAGAAA |
| ATX4 | CTCTGCGATCCACGTAAACA | ATGAGGGGAGGCTTGTTTTT |
| ATX3 | CTGGCAGCCCCAGTATTTT | TTTTCTGGCAGTTGGAATGA |
| ATX1 | GGGTTGAGGGCATCAAATAA | TGTGGCCAATAACAGTGCAT |
